# Aqueductal CSF stroke volume measurements may drive management of shunted idiopathic normal pressure hydrocephalus patients

**DOI:** 10.1038/s41598-021-86350-8

**Published:** 2021-03-29

**Authors:** Antonio Scollato, Saverio Caini, Lucia Angelini, Giancarlo Lastrucci, Nicola Di Lorenzo, Berardino Porfirio, Pasquale Gallina

**Affiliations:** 1 Neurosurgical Unit, Cardinale Panico Hospital, Tricase, Lecce, Italy; 2 Cancer Risk Factors and Lifestyle Epidemiology Unit, Institute for Cancer Research, Prevention, and Clinical Network (ISPRO), Florence, Italy; 3 Department of NEUROFARBA, University of Florence, Florence, Italy; 4 Florence School of Neurosurgery, University of Florence, Florence, Italy; 5 Neurological Centre of Latium, Rome, Italy; 6 Careggi University Hospital, Florence, Italy; 7 Department of Clinical and Experimental Biomedical Sciences “Mario Serio”, University of Florence, Viale Gaetano Pieraccini, 6, 50139 Florence, Italy

**Keywords:** Diseases, Neurological disorders, Hydrocephalus

## Abstract

CSF shunting with adjustable valve is the treatment of idiopathic normal pressure hydrocephalus. The opening pressure valve setting is left to the neurosurgeon’s experience. Aqueductal CSF stroke volume by phase-contrast magnetic resonance measures the CSF passing through the Sylvian aqueduct and it changes with intracranial hydrodynamics. We sought to identify a window of stroke volume differences associated with the best clinical outcome and lowest rate of complications. The records of 69 patients were reviewed. At every clinical check, stroke volume, opening pressure valve, clinical outcome, and CSF overdrainage were analyzed. The correlation between stroke volume differences and negative outcome was also analyzed. The median follow-up was 2.3 years (range 0.3–10.4 years). The odds of negative outcome between two consecutive checks significantly increased by 16% (95%CI 4–28%, *p* = 0.006). Taking the lowest risk group as reference, the odds ratio of negative outcome was 1.16 (95%CI 0.51–2.63, *p* = 0.726) for SV differences less than − 37.6 µL, while it was 1.96 (95%CI 0.97–3.98, *p* = 0.062) for stroke volume changes above + 23.1 µL. Maintaining stroke volume values within a definite range might help maximize clinical benefit and avoid the risk of CSF overdrainage.

## Introduction

Idiopathic normal pressure hydrocephalus (iNPH) is a derangement of intracranial hydrodynamic leading to CSF endo-ventricular accumulation^1,2^. With the boost of arterial pulsation, the brain is compressed against the calvaria, with progressive vascular damage, atrophy, and reduction of ventricular squeezing, which is the force moving CSF^3^. Aqueductal CSF stroke volume (SV) by phase-contrast magnetic resonance measures the volume of the CSF passing through the Sylvian aqueduct under a heart cycle^4^. SV is indicative of brain pulsatility^4^. SV measurement has been proposed in the management of iNPH, as a predictor of CSF shunting outcome^5–7^, although controversially^8–10^. In un-shunted patients, after an increase at the onset of the disease, SV reaches a plateau, then a decline in association with a worsening of the clinical picture, as cerebrovascular damage progresses^11^. In patients receiving ventriculoperitoneal shunt placement with a fixed valve, SV decreases after surgery^12^. Subsequently, there is further decrement related to the brain atrophy, which parallels loss of benefit^12^. A relevant decrease of SV values heralded intracranial fluid collection (IFC), a CSF overdrainage-related complication, which can be corrected by valve closure although at the expense of the clinical benefits^12^.

Adjustable valve allows for non-invasive matching of the opening pressure valve (OPV) with the patient’s intracranial hydrodynamics and clinical course, leading to gains in terms of outcome and treatment of overdrainage complications^13^. However, due to the lack of tools to predict post-shunting deterioration/complications, the physician can only keep track of events and correct them by valve adjustment. Nevertheless, the OPV setting is empirical, which results on the one hand in the risk of an insufficient CSF drainage to obtain a benefit, on the other one in the risk of overdrainage.

Here, we aimed at identifying a range of SV changes achievable by means of OPV resettings during follow-up that might help maximize a patient’s clinical benefit while avoiding overdrainage complications.

## Methods

This study was performed according to the Declaration of Helsinki principles. We reviewed (January–March 2019) the SV changes in relationship with clinical course of a population of iNPH patients who have undergone ventriculoperitoneal shunt placement with adjustable valve (ProGav, Aesculap, Inc or Codman Hakim, Johnson and Johnson). This was a retrospective observational study of already existing data, without any further intervention and a formal institutional review board approval was not mandatory. Data were anonymized before carrying out the analyses. Patients provided consent to use their clinical data for research purposes. iNPH management protocols were reported^14^. Informed consent to clinical procedures was obtained from all patients. iNPH had been diagnosed based on at least two disturbances of the Hakim’s-triad^15^, and Evans ratio > 0.30. Cognitive functions, including orientation, registration, attention, calculation, recall, and language, were evaluated by using the Mini-Mental State Examination (MMSE)^16^ in which the minimum score is zero and the maximum is 30. A score of 23 is indicative of cognitive impairment. Urinary disorders were evaluated by a urinary incontinence scale graded in 4 categories: normal urinary function (grade 0), urgent urination or sporadic incontinence (grade 1), frequent incontinence (grade 2), and complete incontinence (grade 3)^12^. Gait disorders were evaluated by a gait scale graded into 4 categories: normal gait (grade 0); discrete imbalance when turning with short steps, widened base, and occasional falling (grade 1); frequent falls and aid needed for ambulation (grade 2); and impossible gait (grade 3)^12^. Patients were selected for ventriculoperitoneal shunt placement on the basis of improvement after lumbar drainage^14^. A gain by ≥ 2 points combining the urinary and gait scales, or a gain by one point in urinary or gait scales and by ≥ 3 points on the MMSE was considered an improvement.

Inclusion criteria for the present study were at least two SV measurements, absence of other neurological diseases and of perioperative complications. Patients who received other CSF shunting diagnostic/therapeutic procedures were excluded.

Patients’ sex, age at ventriculoperitoneal shunt placement, MMSE, gait and urinary scales scores obtained at any check (i.e. clinical status evaluation, possibly associated with SV measurement and/or OPV resetting) were noted. Patients were categorized as unimproved or improved according to the same criteria used to assess outcome of lumbar drainage. Patients whose clinical status did not change were categorized as worsened. We calculated the time interval and the difference in SV (∆SV) and OPV (∆P) values between each pair of subsequent checks. The occurrence of headache, IFC and shunt malfunctioning were also recorded.

Descriptive analyses were conducted using median and range (for continuous variables) and contingency tables (for categorical variables), whereas differences in distribution were tested using the Wilcoxon rank-sum test and the chi-square test. Missing data were imputed through a multiple imputation by chained equations procedure, using truncated linear regression for continuous variables and logistic regression for binary variables. For each patient, we separately considered the first post-shunt SV measurement, to relate the patients’ clinical benefit to the achieved ∆SV, and all subsequent measurements, to identify the ∆SV associated with the best outcome, while minimizing the risk of overdrainage. We used generalized estimating equations to study the relationship between ∆SV (independent variable) and the outcome (dependent variable), adjusting for relevant covariates (checks in which a shunt malfunctioning was detected were not used)^17^. Alternative models were fitted in which ∆SV was entered either as absolute or percentage value, and in which the dependent variable was the clinical status, the presence of overdrainage complications (IFC and/or headache). ∆SV was initially modelled in quintiles and deciles; afterwards, deciles of ∆SV were empirically merged into broader groups to maximize contrasts and identify, in a data-driven way, the range of ∆SV associated with the highest likelihood of positive outcome. Covariates adjusted for in the models were the patient’s sex, age, and baseline MMSE, the baseline SV value and that measured at the previous check, and the number of checks that had taken place until that point. The statistical analyses were conducted using Stata Software Release 14. Tests were two-tailed and considered as significant when *p*-value < 0.05.

## Results

Sixty-nine patients (48 males) met the selection criteria. Four-hundred-sixty-two checks were performed with 355 SV measurements. Median patients age was 76 years (range 67–86 years); median baseline MMSE value was 23 (range 12–28); median follow-up was 2.3 years (range 0.3–10.4 years). Demographic and clinical details of the patients are in Supplementary Information (SI) 1.

### Baseline and follow-up SV and OPV data

OPV value at surgery was 110 mmH_2_O in 56 patients (81.2%), 120 mmH_2_O in 12 (17.4%), 130 mmH_2_O in one (1.4%). Sixty patients (SI 1) underwent baseline SV measurement (median 100.5 µL, range 35–300 µL). The median ∆SV was − 9 µL (range − 123, + 85 µL) between the first check and the baseline, and − 16 µL (range – 150, + 109 µL) between the last check and the baseline.

Eight patients did not undergo OPV resetting. In the remaining 61 patients, the median number of resettings was two (range 1–11), with the median ∆P between the last OPV resetting and the OPV setting at surgery equal to − 10 mmH_2_O (range − 40, + 80 mmH_2_O). The median interval time between two consecutive OPV resettings was 0.89 years (range 0.3–8.7). See Table 1*.*

**Table 1:** Imaging and opening pressure valve data at baseline and during follow-up in 69 idiopathic normal pressure hydrocephalus patients who underwent ventriculoperitoneal shunt placement with adjustable valve.

n. of clinical checks	462	
n. of SV measurements	355	
	Median	Range
SV baseline in µL	100.5	35–300
∆SV at first post-surgical check in µL	− 9 (− 13.7%)	− 123, + 85 (− 84.1%, + 138%)
∆SV at last check in µL	− 16 (− 29.7%)	− 150, + 109 (− 85%, + 107%)
OPV value at VPSp in mmH_2_O	110 in 56 pts 120 in 12 pts 130 in 1 pt	
∆P at last check in mmH_2_O	− 10	− 40, − 80
n. OPV resettings/pt	2	1–11

*n.* number; *OPV* opening pressure valve; *pt(s)* patient(s); *SV* aqueductal cerebrospinal fluid stroke volume; *VPSp* ventriculoperitoneal shunt placement.

Clinical check indicates the clinical evaluation, possibly associated with SV measurement and/or OPV resetting; ∆SV indicates the difference between SV values at baseline and during follow-up; ∆P indicates the difference of OPV setting at surgery and during follow-up.

### Baseline and follow-up SV and OPV data in the patients who experienced overdrainage-related complications and comparisons with non-complicated ones

Twenty-five patients experienced complications, of whom 14 had only headache, nine had only IFC, and two had both headache and IFC.

Among the eleven patients who experienced IFC, OPV value at the occurrence of IFC ranged between 110 and 40 mmH_2_O. The median interval time from surgery to IFC was 1.9 years (range: 0.2–6.2). After IFC, OPV was increased by 30 to 70 mmH_2_O. Patients who experienced IFC had more checks than those who did not (median 9 vs. five, *p* = 0.003), while there were no differences in terms of age, gender and MMSE. The median ∆SV was − 33 µL when IFC occurred, and − 4 µL when it did not (*p* < 0.001) (Table 2).

**Table 2:** Aqueductal cerebrospinal fluid stroke volume changes, opening pressure valve data during follow-up, in 11 cerebrospinal fluid shunted patients with idiopathic normal pressure hydrocephalus who experienced intracranial fluid collection and comparison with no complicated patients.

n. events/pt	2 in 1pt, 1 in 10 pts				
OPV value in mmH2O at IFC	110 in 2 pts, 90 in 1 pt, 70 in 1 pt, 60 in 3 pts, 40 in 2 pts				
	Median	Range			
∆T from VPS to IFC (years)	1.9	0.2–6.2			

	pts who experienced IFC		pts who did not experience IFC		*p* value
	Median	Range	Median	Range	
n. checks/pt	9	(1–17)	5	(1–10)	0.003
% of clinical checks where OPV resetting were performed	54.5%	(95%CI 44.2–64.4)	45.2%	(95%CI 39.4–51.1)	0.109
∆SV in µL	− 33	− 80, + 1	− 4	− 135, + 108	< 0.001
	(− 25.4%)	(− 45.7%, + 1.2%)	(− 3.8%)	(− 88%, + 560%)	

*CI* confidence interval, ∆T time interval, *IFC* intracranial fluid collection, *n*. number, *OPV* opening pressure valve, *pt(s)* patient(s), *VPSp* ventriculo-peritoneal shunt placement.

Clinical check indicates the clinical evaluation possibly associated with aqueductal cerebrospinal fluid stroke volume (SV) measurement and/or OPV resetting; in IFC patients ∆SV indicates the difference between SV values from the check where IFC was detected and the previous one, while in patients who did not experience IFC indicates the median of SV changes during follow-up.

Among the sixteen patients who experienced headache, OPV value at the occurrence ranged between 160 and 30 mmH_2_O. Headache occurred at a median of 0.4 years after surgery (range 0.2–9 years), and resolved in all patients upon increasing the OPV value by 10 to 50 mmH_2_O. Patients characteristics (gender, age, MMSE, number of checks and resettings) did not differ between patients who experienced headache compared to those who did not.

### SV changes in patients who experienced valve malfunctioning

Fourteen patients experienced shunt malfunctioning; the shunt system was replaced in all. Seven patients received SV measurement at the check when the malfunction was recognized. In two of these, the malfunction was heralded by an increase of SV (from 94 to 135 µL, and from 144 to 189 µL); in two others, SV remained stable despite OPV resettings. In three cases, SV presented marginal changes.

### Risk of worsening of Hakim’s-triad and of overdrainage complications related to ∆SV

The risk of worsening of Hakim’s-triad had a rising trend as quintiles progressed. Particularly, the OR of a worsening of Hakim’s-triad was increased by over 80% for ∆SV above + 13.1 µL see SI 2. The contrast was maximized (and achieved statistical significance) by comparing deciles 7–10 versus 1–6 of the distribution of ∆SV: the OR for the comparison of the former vs. the latter was 1.86 (95%CI 1.03–3.38, *p* = 0.041). The risk of worsening of Hakim’s-triad increased significantly at each subsequent check (OR 1.15, 95%CI 1.05–1.28, *p* 0.005), see SI 3. No associations were found with patients’ age, sex, and baseline MMSE or SV.

The results of the same analysis conducted using the occurrence of overdrainage complications as outcome were shown in SI 4. The lowest risk of overdrainage was observed for the deciles from the fourth to the ninth (corresponding to the range from − 22.4 to + 61.0 µL) in the distribution of ∆SV. Taking this category as a reference, the OR was 2.23 (95%CI 0.85–5.83, *p* = 0.101) for the lowest deciles (from first to third, range from − 246.9 to − 22.4 µL), and 2.34 (95%CI 0.40–13.84, *p* 0.348) for the top decile of the distribution (from + 61.0 to + 253.8 µL) (SI 5). The OR for females was 2.53 (95%CI 0.78–8.21, *p* 0.123); no association with the others parameters was found.

**Figure 1:**
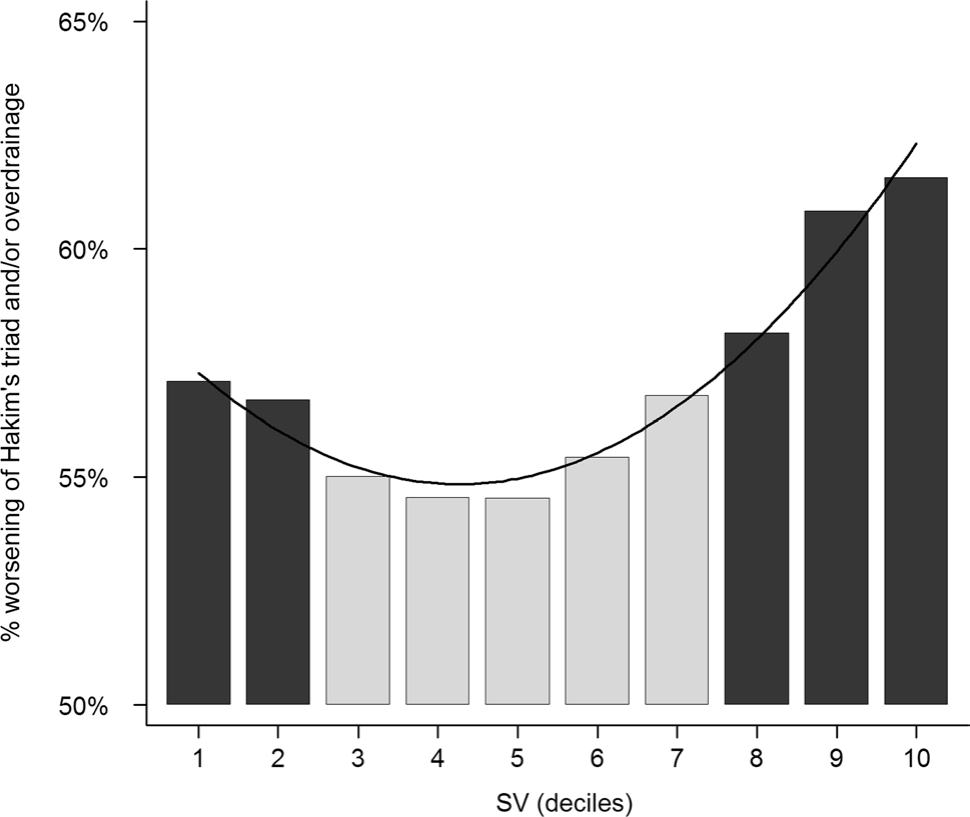
Histogram of the risk of clinical worsening and occurrence of cerebrospinal fluid overdrainage complications as deciles of difference in aqueductal cerebrospinal fluid stroke volume (∆SV). ∆SV indicates the difference between aqueductal cerebrospinal fluid stroke volume values at any clinical check and the previous one. ∆SV deciles corresponding to maximum risk are shown in black.

We finally considered as the outcome the risk of having either a worsening of Hakim’s-triad or an overdrainage complication (results in SI 6). The risk was lowest for the deciles in the ∆SV distribution from the third to the seventh (corresponding to ∆SV ranging between − 37.6 and + 23.0 µL). Taking this as a reference, the OR for this composite outcome was 1.16 (95%CI 0.51–2.63, *p* 0.726) for the two lower deciles (∆SV from − 246.9 to − 37.6 µL), and 1.96 (95%CI 0.97–3.98, *p* 0.062) in the upper three deciles (from + 23.1 to + 253.8 µL) (SI 7 and Fig. 1). The OR increased between each pair of consecutive checks (1.16, 95%CI 1.04–1.28, *p* 0.006) and was higher (close to statistical significance) among females (1.80, 95%CI 0.93–3.48, *p* 0.079); no other parameter was associated with significant results.

## Discussion

SV decreased of a median 13.7% after shunting due to the opening of an alternative way for the CSF flow associated with clinical benefit in most of patients (94%). The possibility of changing the OPV makes it possible to counteract the loss of benefit with the progression of the disease, at the expense of the risk of overdrainage. However, SV continues to decrease due to the course of degeneration phenomena and the reduction of cerebral pulsatility which will lead to a “point of no return”, i.e. the moment when the correlation between the amount of drained CSF, SV trend and clinical outcome will be lost. The risk of worsening of Hakim’s-triad and occurrence of overdrainage increased significantly by 16% between one check and the following one. Since the “point of no return” is unknown (due to the limited number of patients with adequately long follow-up), we sought to identify a window of ∆SV values that maximized clinical benefits and minimized the risk of overdrainage. To do so, we ordered all checks, irrespective of the patients, according to ∆SV values and taking into account the longitudinal nature of the data (with patients contributing a varying number of measurements over time to the study database).

Overdrainage complications were heralded by ∆SV lower than − 22.4 µL (OR 2.23, *p* 0.101) without correlation with time after shunting and, therefore, with the progression of brain degeneration. While statistical significance was not achieved (possible because of the limited sample size), the strength of the association and the solid scientific rationale suggest that the observed association might be real. These complications are iatrogenic, most likely consequent by the lack of clear guidance of the values at which the valve has to be reset. A risk of overdrainage complications was found also for ∆SV upper than + 61.0 µL (OR 2.34). While a chance finding may not be excluded (the *p*-value for this association was 0.348), this paradoxical result might be the consequence of our having grouped together IFC and headache, which may indeed be caused by overdrainage but also have other causes. Particularly, headache was self-reported and not investigated specifically, which may have led to some misclassification. These considerations are strengthened by the finding of a non-significantly, but substantially higher risk of overdrainage complications among females (OR 2.53, *p* 0.123), who suffer from headaches more than males^18^. Further investigations taking advantage of an accurate definition of overdrainage-related headache might lead to a larger ∆SV window i.e. more room to pursue clinical benefit.

The association between better patient’s outcome and greater baseline SV values is known^7,12^. However, since outcome can be improved until the “point of no-return” is reached, SV baseline value should not be used as a criterion to exclude patients from shunting. Prospective studies might stratify patient’s baseline SV value associated with duration and intensity of clinical improvement. In patients with relatively low baseline SV values, the balance between surgical risks^19^ and the entity of benefit will drive the decision. Baseline SV value could be used to choose OPV setting at surgery. In clinical practice, the surgical valve setting is standard, mostly at 110 or 120 mmH_2_O^13^. In patients with severe cerebral atrophy, this setting may not be sufficient to make the valve work efficiently. In patients with a fixed valve, this results in a negative outcome from the beginning; in patients with adjustable valve, instead, the possibility to find the best post-shunt OPV setting exists, but to date, this optimal value is searched only through reiterated empirical OPV resettings.

Extreme rises of SV values forewarn shunt malfunctioning^12^. Here, the rise of SV values may just lead to suspect malfunctioning. A tendency for SV to remain stable while neurological status does not improve despite OPV downward resetting should also lead to a suspicion of shunt malfunctioning.

This study presents limitations. The checks were not performed at regular intervals and their frequency was sometimes determined based on the patients’ clinical condition. SV was not systematically measured, and the outcome was not assessed, at every check. This imposed the need for a multiple imputation to be carried out to enhance the reliability of the results. Overall, limitations did not affect the methodological robustness of analytical procedures that were adopted. Particularly, our methodology took into account the potential misclassification of patients’ outcome mentioned above and the study size possibly not sufficient to detect clinically relevant, yet moderate changes (e.g. by less than 50%) in the frequency of negative outcome. Therefore, our study showed clearly discernible and biologically plausible associations and trends between ∆SV values and outcome. While the trend in the association between ∆SV values and patient’s symptoms seems convincing, the optimal cut-offs we identified may be specific of our population.

## Conclusions

Upon confirmation from future studies with a larger population size and more accurate definition of outcomes (which would lead to greater statistical power and thus clearer results), the ∆SV window may permit, by either up or down OPV regulation leading to small ∆SV, to optimize the clinical course of shunted patients and extend the spectrum of patients who may benefit from shunting. The availability of an accurate prognostic test^7,14^, to exclude from surgery non-hydrocephalic patients, and a range of ∆SV values within which the clinical outcome is maximized, may provide relief to patients regardless of their degree of brain atrophy. Stratification of patients according to their intracranial hydrodynamics, hence with different optimal ∆SV windows, might allow a better outcome within each subgroup. Occurrence of post-shunting hypoacusis^20^ and glaucoma^21^, should also be taken into account when determining the optimal ∆SV window. Once the correlation between OPV values and the correspondent ∆SV is understood, it would be worth design adjustable valves having a greater capacity for fine-tuning.

## Supplementary Information

Supplementary Information 1.

Supplementary Information 2.

Supplementary Information 3.

Supplementary Information 4.

Supplementary Information 5.

Supplementary Information 6.

Supplementary Information 7.

## Data Availability

The datasets generated and/or analysed during the current study are not publicly available due to participant’s privacy protection but are available from the corresponding author on reasonable request.
