# Supplementary material for: Aqueductal CSF stroke volume measurements may drive management of shunted idiopathic normal pressure hydrocephalus patients
Source: Sci Rep. 2021 Mar 29;11:7095. doi: 10.1038/s41598-021-86350-8 (PMC8007697; doi:10.1038/s41598-021-86350-8)
Supplement: Supplementary file 1 — Supplementary Information 1. [file 41598_2021_86350_MOESM1_ESM.docx]

Aqueductal CSF stroke volume measurements may drive management of shunted idiopathic normal pressure hydrocephalus patients

Antonio Scollato, MD,^1^ Saverio Caini, MD,^2^ Lucia Angelini, MD,^3^ Giancarlo Lastrucci, MD,^3,4^ Nicola Di Lorenzo, MD,^5^ Berardino Porfirio, MD,*^6,7^ Pasquale Gallina, MD^3,4,6^

- 1) Neurosurgical Unit, Cardinale Panico Hospital, Tricase, Lecce, Italy
- 2) Cancer Risk Factors and Lifestyle Epidemiology Unit, Institute for Cancer Research, Prevention, and Clinical Network (ISPRO), Florence, Italy
- 3) Department of NEUROFARBA, University of Florence, Italy
- 4) Florence School of Neurosurgery, University of Florence, Italy
- 5) University of Florence, Italy
- 6) Careggi University Hospital, Florence, Italy
- 7) Department of Clinical and Experimental Biomedical Sciences “Mario Serio”, University of Florence, Italy

**Table 1. Demographic, and baseline clinical characteristics and aqueductal CSF stroke volume values in a series of idiopathic normal pressure hydrocephalus patients who underwent ventriculoperitoneal shunt with adjustable valve**

| **Patient** | **Sex** | **Age** | **MMSE score** | **Gait score** | **Urinary score** | **Stroke volume** (**μL)** |
| --- | --- | --- | --- | --- | --- | --- |
| 1 | M | 83 | 20 | 2 | 2 | 86 |
| 2 | M | 77 | 25 | 2 | 2 | 67 |
| 3 | M | 77 | 26 | 2 | 1 | 109 |
| 4 | M | 78 | 25 | 2 | 1 |  |
| 5 | F | 79 | 25 | 2 | 2 |  |
| 6 | M | 80 | 27 | 2 | 1 | 199 |
| 7 | M | 75 | 23 | 2 | 2 |  |
| 8 | F | 79 | 19 | 2 | 0 | 100 |
| 9 | M | 79 | 12 | 1 | 0 | 178 |
| 10 | M | 80 | 24 | 2 | 3 | 80 |
| 11 | M | 70 | 23 | 2 | 2 | 80 |
| 12 | F | 76 | 25 | 2 | 2 | 132 |
| 13 | M | 86 |  | 2 | 2 | 83 |
| 14 | F | 76 | 27 | 2 | 2 | 102 |
| 15 | F | 73 | 15 | 2 | 3 | 130 |
| 16 | M | 75 | 25 | 1 | 2 | 101 |
| 17 | M | 80 | 20 | 2 | 2 | 63 |
| 18 | M | 83 | 28 | 2 | 1 | 110 |
| 19 | F | 82 | 16 | 2 | 2 | 54 |
| 20 | M | 84 | 25 | 2 | 2 | 89 |
| 21 | M | 77 | 12 | 3 | 2 | 105 |
| 22 | M | 82 | 23 | 2 | 1 | 70 |
| 23 | M | 77 | 23 | 2 | 2 |  |
| 24 | F | 78 | 16 | 3 | 3 | 104 |
| 25 | M | 73 | 27 | 2 | 2 |  |
| 26 | F | 80 | 24 | 2 | 2 | 133 |
| 27 | M | 70 | 23 | 2 | 2 | 65 |
| 28 | F | 73 | 27 | 1 | 2 | 100 |
| 29 | M | 75 | 23 | 1 | 2 |  |
| 30 | M | 69 | 23 | 3 | 2 | 76 |
| 31 | F | 78 |  | 2 | 2 |  |
| 32 | M | 74 | 19 | 3 | 3 | 115 |
| 33 | F | 81 | 23 | 2 | 0 | 50 |
| 34 | M | 80 | 21 | 2 | 2 | 63 |
| 35 | M | 86 | 28 | 2 | 0 | 233 |
| 36 | M | 75 | 28 | 1 | 2 | 260 |
| 37 | F | 82 | 19 | 3 | 2 | 100 |
| 38 | M | 76 |  | 2 | 1 | 67 |
| 39 | M | 73 | 27 | 3 | 1 | 35 |
| 40 | M | 85 | 19 | 2 | 1 | 44 |
| 41 | M | 72 | 27 | 1 | 1 | 160 |
| 42 | F | 79 | 26 | 2 | 2 |  |
| 43 | M | 77 | 28 | 2 | 1 | 119 |
| 44 | M | 69 | 28 | 1 | 2 | 88 |
| 45 | M | 75 | 21 | 2 | 2 | 89 |
| 46 | M | 76 | 22 | 2 | 2 | 126 |
| 47 | M | 79 | 27 | 1 | 2 | 133 |
| 48 | M | 75 | 26 | 1 | 0 | 57,5 |
| 49 | M | 83 | 16 | 2 | 1 | 44 |
| 50 | M | 74 | 24 | 2 | 1 | 91 |
| 51 | F | 72 |  | 2 | 0 | 128 |
| 52 | F | 76 | 17 | 1 | 1 | 166 |
| 53 | M | 76 | 17 | 2 | 2 | 129 |
| 54 | F | 75 | 25 | 1 | 2 | 150 |
| 55 | M | 70 | 24 | 2 | 1 | 300 |
| 56 | M | 77 | 17 | 2 | 1 | 235 |
| 57 | M | 75 | 19 | 2 | 3 | 50 |
| 58 | M | 67 | 22 | 1 | 2 | 151 |
| 59 | F | 75 | 15 | 1 | 2 | 177 |
| 60 | F | 74 | 25 | 1 | 1 | 44 |
| 61 | M | 85 | 25 | 1 | 2 | 54 |
| 62 | M | 76 | 16 | 2 | 1 | 147 |
| 63 | F | 84 | 25 | 2 | 3 | 100 |
| 64 | M | 75 | 16 | 3 | 3 |  |
| 65 | F | 73 | 17 | 3 | 3 | 156 |
| 66 | M | 77 | 26 | 1 | 2 | 145 |
| 67 | M | 73 | 24 | 1 | 1 | 95 |
| 68 | M | 80 | 25 | 1 | 1 | 223 |
| 69 | F | 71 | 23 | 2 | 3 | 62 |

CSF: cerebrospinal fluid; M: male, F: female; MMSE: Mini-Mental State Examination.

Cognitive functions, including orientation, registration, attention, calculation, recall, and language, were evaluated by using the MMSE,^16^ in which the minimum score is zero and the maximum is 30. A score of 23 is indicative of cognitive impairment. Urinary disorders were evaluated by a urinary incontinence scale graded in 4 categories: normal urinary function (grade 0), urgent urination or sporadic incontinence (grade 1), frequent incontinence (grade 2), and complete incontinence (grade 3).^12^ Gait disorders were evaluated by a gait scale graded into 4 categories: normal gait (grade 0); discrete imbalance when turning with short steps, widened base, and occasional falling (grade 1); frequent falls and aid needed for ambulation (grade 2); and impossible gait (grade 3).^12^
