# Supplementary material for: Aqueductal CSF stroke volume measurements may drive management of shunted idiopathic normal pressure hydrocephalus patients
Source: Sci Rep. 2021 Mar 29;11:7095. doi: 10.1038/s41598-021-86350-8 (PMC8007697; doi:10.1038/s41598-021-86350-8)
Supplement: Supplementary file 2 — Supplementary Information 2. [file 41598_2021_86350_MOESM2_ESM.docx]

Aqueductal CSF stroke volume measurements may drive management of shunted idiopathic normal pressure hydrocephalus patients

Antonio Scollato, MD,^1^ Saverio Caini, MD,^2^ Lucia Angelini, MD,^3^ Giancarlo Lastrucci, MD,^3,4^ Nicola Di Lorenzo, MD,^5^ Berardino Porfirio, MD,*^6,7^ Pasquale Gallina, MD^3,4,6^

- 1) Neurosurgical Unit, Cardinale Panico Hospital, Tricase, Lecce, Italy
- 2) Cancer Risk Factors and Lifestyle Epidemiology Unit, Institute for Cancer Research, Prevention, and Clinical Network (ISPRO), Florence, Italy
- 3) Department of NEUROFARBA, University of Florence, Italy
- 4) Florence School of Neurosurgery, University of Florence, Italy
- 5) University of Florence, Italy
- 6) Careggi University Hospital, Florence, Italy
- 7) Department of Clinical and Experimental Biomedical Sciences “Mario Serio”, University of Florence, Italy

**Table 2. Association between changes in the aqueductal stroke volume changes (modeled in quintiles) and the risk of stable negative clinical outcome (worsening of Hakim’s triad^1^)**

| Predictor | OR | Lower 95%CI | Upper 95%CI | p-value | SV ranges | |
| --- | --- | --- | --- | --- | --- | --- |
| ∆SV (abs, quintiles) |  |  |  |  | *min* | *max* |
| 1 | ref |  |  |  | -246.9 | -37.6 |
| 2 | 1.04 | 0.40 | 2.68 | 0.93 | -3.6 | -9.0 |
| 3 | 1.13 | 0.43 | 2.97 | 0.811 | -9.0 | +13.1 |
| 4 | 1.82 | 0.65 | 5.09 | 0.251 | +13.1 | +38.0 |
| 5 | 2.23 | 0.73 | 6.78 | 0.158 | +38.0 | +253.8 |
| Female sex | 1.26 | 0.61 | 2.58 | 0.527 |  |  |
| Round (+1) | 1.16 | 1.05 | 1.28 | 0.005 |  |  |
| MMSE baseline |  |  |  |  |  |  |
| ≥25 | ref |  |  |  |  |  |
| 19-24 | 0.98 | 0.48 | 1.99 | 0.951 |  |  |
| ≤18 | 0.88 | 0.40 | 1.93 | 0.755 |  |  |
