# Supplementary material for: Aqueductal CSF stroke volume measurements may drive management of shunted idiopathic normal pressure hydrocephalus patients
Source: Sci Rep. 2021 Mar 29;11:7095. doi: 10.1038/s41598-021-86350-8 (PMC8007697; doi:10.1038/s41598-021-86350-8)
Supplement: Supplementary file 5 — Supplementary Information 5. [file 41598_2021_86350_MOESM5_ESM.docx]

Aqueductal CSF stroke volume measurements may drive management of shunted idiopathic normal pressure hydrocephalus patients

Antonio Scollato, MD,^1^ Saverio Caini, MD,^2^ Lucia Angelini, MD,^3^ Giancarlo Lastrucci, MD,^3,4^ Nicola Di Lorenzo, MD,^5^ Berardino Porfirio, MD,*^6,7^ Pasquale Gallina, MD^3,4,6^

- 1) Neurosurgical Unit, Cardinale Panico Hospital, Tricase, Lecce, Italy
- 2) Cancer Risk Factors and Lifestyle Epidemiology Unit, Institute for Cancer Research, Prevention, and Clinical Network (ISPRO), Florence, Italy
- 3) Department of NEUROFARBA, University of Florence, Italy
- 4) Florence School of Neurosurgery, University of Florence, Italy
- 5) University of Florence, Italy
- 6) Careggi University Hospital, Florence, Italy
- 7) Department of Clinical and Experimental Biomedical Sciences “Mario Serio”, University of Florence, Italy

| **Table 5. Association between changes in the aqueductal cerebrospinal fluid stroke volume changes (modeled in quintiles) and the risk of negative clinical outcome (worsening of Hakim’s triad^1^) and/or cerebrospinal fluid overdrainage complications (intracranial fluid collection and headache)** | | | | | | |
| --- | --- | --- | --- | --- | --- | --- |
|  |  |  |  |  |  |  |
|  |  |  |  |  |  |  |
| predictor | OR | Lower 95%CI | Upper 95%CI | p-value | SV changes | |
| ∆SV (abs) |  |  |  |  | *min* | *max* |
| quintiles |  |  |  |  |  |  |
| 1 | ref |  |  |  | -246.9 | -37.6 |
| 2 | 0.91 | 0.33 | 2.53 | 0.854 | -37.6 | -9.0 |
| 3 | 0.85 | 0.32 | 2.26 | 0.745 | -9.0 | +13.1 |
| 4 | 1.02 | 0.35 | 2.95 | 0.972 | +13.1 | +38.0 |
| 5 | 1.00 | 0.30 | 3.34 | 0.996 | +38.0 | +253.8 |
| Female sex | 2.00 | 1.06 | 3.77 | 0.032 |  |  |
| Round (+1) | 1.13 | 1.03 | 1.25 | 0.009 |  |  |
| MMSE baseline |  |  |  |  |  |  |
| ≥25 | ref |  |  |  |  |  |
| 19-24 | 1.00 | 0.51 | 1.96 | 0.992 |  |  |
| ≤18 | 1.08 | 0.51 | 2.30 | 0.834 |  |  |
| abs = absolute; CI = confidence interval; MMSE = mini-mental-state examination;^16^ OR = odd ratio; ref = reference; SV = aqueductal cerebrospinal fluid stroke volume; ∆SV indicates the difference of SV values between a clinical check and the previous one; Round (+1) indicates the difference between a clinical check and the previous one. | | | | | | |
|  |  |  |  |  |  |  |
|  |  |  |  |  |  |  |
|  |  |  |  |  |  |  |
