# Supplementary material for: Aqueductal CSF stroke volume measurements may drive management of shunted idiopathic normal pressure hydrocephalus patients
Source: Sci Rep. 2021 Mar 29;11:7095. doi: 10.1038/s41598-021-86350-8 (PMC8007697; doi:10.1038/s41598-021-86350-8)
Supplement: Supplementary file 6 — Supplementary Information 6. [file 41598_2021_86350_MOESM6_ESM.pdf]

Aqueductal CSF stroke volume measurements may drive management of shunted idiopathic normal pressure hydrocephalus patients

Antonio Scollato, MD,[1] Saverio Caini, MD,[2] Lucia Angelini, MD,[3] Giancarlo Lastrucci, MD,[3,4] Nicola Di Lorenzo, MD,[5] Berardino Porfirio, MD,*[6,7] Pasquale Gallina, MD[3,4,6]

1) Neurosurgical Unit, Cardinale Panico Hospital, Tricase, Lecce, Italy

2) Cancer Risk Factors and Lifestyle Epidemiology Unit, Institute for Cancer Research, Prevention, and Clinical Network (ISPRO), Florence, Italy

3) Department of NEUROFARBA, University of Florence, Italy

4) Florence School of Neurosurgery, University of Florence, Italy

5) University of Florence, Italy

6) Careggi University Hospital, Florence, Italy

7) Department of Clinical and Experimental Biomedical Sciences "Mario Serio", University of Florence, Italy

Table 6. Association between changes in the aqueductal cerebrospinal fluid stroke volume changes (modeled in quintiles) and the risk of stable or negative clinical outcome and/or cerebrospinal fluid overdrainage complications (intracranial fluid collection and headache)

predictor	OR	Lower 95%CI	Upper 95%CI	p-value	SV changes	
ΔSV (abs)					*min*	*max*
quintiles						
1	ref				-246.9	-37.6
2	0.82	0.31	2.15	0.681	-37.6	-9.0
3	0.81	0.32	2.07	0.665	-9.0	+13.1
4	1.25	0.45	3.48	0.675	+13.1	+38.0
5	1.77	0.58	5.37	0.310	+38.0	+253.8
Female sex	1.83	0.95	3.55	0.073		
Round (+1)	1.16	1.04	1.28	0.005		
MMSE baseline						
≥25	ref					
19-24	1.24	0.64	2.41	0.529		
≤18	0.84	0.38	1.87	0.672		

abs = absolute; CI = confidence interval; MMSE = mini-mental-state examination;[16] OR = odd ratio; ref = reference; SV = aqueductal cerebrospinal fluid stroke volume; ΔSV indicates the difference of SV values between a clinical check and the previous one; Round (+1) indicates the difference between a clinical check and the previous one.
